# 25-Hydroxy-Vitamin D Concentration Is Not Affected by Severe or Non-Severe Pneumonia, or Inflammation, in Young Children

**DOI:** 10.3390/nu9010052

**Published:** 2017-01-17

**Authors:** Johanne Haugen, Ram K. Chandyo, Manjeswori Ulak, Maria Mathisen, Sudha Basnet, Karl A. Brokstad, Palle Valentiner-Branth, Prakash S. Shrestha, Tor A. Strand

**Affiliations:** 1 Innlandet Hospital Trust, Lillehammer, Anders Sandvigs gt. 17, 2609 Lillehammer, Norway; tors@me.com; 2 Centre for Intervention Science in Maternal and Child Health, Centre for International Health, University of Bergen, Årstav. 21, 5009 Bergen, Norway; 3 Department of Community Medicine, Kathmandu Medical College, P.O. Box 21266, Sinamangal, 44600 Kathmandu, Nepal; ram.chandyo@cih.uib.no; 4 Department of Child Health, Institute of Medicine, Tribhuvan University, Maharajgunj, P.O. Box 1524, 44600 Kathmandu, Nepal; manjeswori@gmail.com (M.U.); sudhacbasnet@gmail.com (S.B.); prakashsunder@hotmail.com (P.S.S.); 5 University Hospital of Northern Norway, Sykehusv. 38, 9019 Tromsø, Norway; mathisen.maria@gmail.com; 6 Broegelmann Research Laboratory, Dept. of Clinical Sciences, University of Bergen, Jonas Lies v. 87, 5021 Bergen, Norway; karl.brokstad@k2.uib.no; 7 Department of Infectious Disease Epidemiology, Statens Serum Institut, Artilleriv 5, 2300 Copenhagen S, Denmark; pvb@ssi.dk

**Keywords:** acute lower respiratory tract infection, children, inflammation, vitamin D, Nepal

## Abstract

Poor vitamin D status has been associated with increased risk and severity of respiratory tract infections. Whether or not inflammation and infection affects 25-hydroxy vitamin D (25(OH)D) concentration is controversial and is important in the interpretation of observational studies using plasma-25(OH)D as a biomarker for status. Our objectives were to measure whether 25(OH)D concentration was altered by an episode of acute lower respiratory tract infection and whether markers of inflammation predicted the 25(OH)D concentration. Children aged 2–35 months with severe (*n =* 43) and non-severe (*n =* 387) community-acquired, WHO-defined pneumonia were included. 25(OH)D concentration and inflammatory markers (cytokines, chemokines, and growth factors) were measured in plasma during the acute phase and 14, 45, and 90 days later. Predictors for 25(OH)D concentrations were identified in multiple linear regression models. Mean 25(OH)D concentration during the acute phase and after recovery (14, 45, and 90 days) was 84.4 nmol/L ± 33.6, and 80.6 ± 35.4, respectively. None of the inflammatory markers predicted 25(OH)D concentration in the multiple regression models. Age was the most important predictor for 25(OH)D concentration, and there were no differences in 25(OH)D concentrations during illness and after 14, 45, and 90 days when adjusting for age. Infection and inflammation did not alter the 25(OH)D concentration in young children with acute lower respiratory tract infections.

## 1. Introduction

Several observational studies have reported an association between vitamin D status and the incidence and severity of respiratory tract infections in children [1,2,3,4]. Despite demonstration of several immune-modulating effects of vitamin D/vitamin D receptor signaling, such as transcription of anti-microbial compounds and regulation of cytokine production and immune cell activity [5,6,7,8,9], the mechanisms underlying these relationships are still not fully understood. The associations have also been difficult to reproduce in clinical trials, which is reflected in the heterogeneous results from recent meta-analyses of this field [10,11,12]. Additionally, there are examples from studies showing reduced 25(OH)D concentration during acute episodes of illness/inflammation [13,14,15], which has prompted the hypothesis that vitamin D concentration may be affected by inflammation and infection. Recently, the authors of a systematic review concluded that vitamin D status measured during the acute phase should be interpreted with care [16], but most of the studies included in this systematic review were in non-infectious patients, except for one study including 14 adult patients with malaria, in whom no change in 25(OH)D was found between the acute phase and a follow-up 2–6 weeks later [17]. In another recent study of 30 children aged between nine months and 16 years with bacterial illnesses, four of whom had pneumonia, no significant difference was found in the 25(OH)D concentration during the illness compared to after one month [18]. We are not aware of similar studies in younger children with acute lower respiratory tract infection (ALRI).

Confirmation of the hypothesis of 25(OH)D as a possible negative acute phase reactant in respiratory tract infections could call for changes in the way we interpret the results and the observed associations between vitamin D status and illness. In the present study of 430 Nepalese children, aged 2–35 months, with WHO-defined non-severe and severe pneumonia, we wanted to address this question further by measuring 25(OH)D concentration during the illness and after recovery and by identifying predictors of 25(OH)D concentration.

## 2. Materials and Methods

### 2.1. Study Population and Study Design

In this study we are using plasma samples from a previously completed randomized, double-blind, placebo-controlled trial (RCT) in Bhaktapur, Nepal (clinicaltrials.gov: NCT00148733) [19], the primary objective of which was to measure the clinical effect of zinc supplementation in children with ALRI (WHO-defined severe and non-severe pneumonia) [20]. A secondary objective was to measure the vitamin D status in this population. Patients were enrolled from June 2004 to June 2007, and out of a study sample of 2628, 430 were randomly selected for zinc, vitamin status, and inflammatory profile analysis. The acute inflammatory profiles in non-severe and severe pneumonia groups have previously been described in a separate publication [21]. The study population consisted of children with pneumonia, attending the study clinic at Siddhi Memorial Hospital (in Bhaktapur) because of cough and/or difficulty breathing. Non-severe pneumonia was diagnosed by the criteria of cough or breathing difficulties and age-adjusted tachypnea (for children aged 2–11 months defined as ≥50 breaths/min and for children aged ≥12 months defined as ≥40 breaths/min). The presence of lower chest indrawing (LCI) or other danger signs (such as inability to eat or drink, vomiting, convulsions, lethargy, or unconsciousness) was used to define severe pneumonia. Children with wheezing were given two doses of nebulized salbutamol and reassessed after 30 min to see whether he or she still fulfilled the inclusion criteria. Lack of consent, not planning to live in the area for the next six months, requiring care for very severe disease, severe malnutrition (defined as being <70% of the median weight for height according to National Centre for Health Statistics standards), the presence of congenital heart disease, documented tuberculosis, documentation of any oral antibiotic treatment in the past 48 h, cough for >14 days, severe anemia (defined as hemoglobin <7 g/dL), and dysentery were exclusion criteria. Children with severe pneumonia were admitted to the hospital for parenteral treatment with benzyl penicillin and other supportive treatment. The day of recovery from severe pneumonia was defined as the beginning of the first 24-h period without LCI, without grunting, and with no nasal flaring. Antibiotics were then changed to oral amoxicillin for a total of five more days, and the child would normally be discharged from hospital and monitored daily by a field worker until recovery from non-severe pneumonia. Children with non-severe pneumonia were treated with oral cotrimoxazole according to weight twice daily for five days with a daily follow-up of field workers visiting the child and the parents in their home until recovery. Recovery from non-severe pneumonia was defined as the first of two consecutive days with a normal respiratory rate.

### 2.2. Data Collection and Laboratory Procedures

The procedures of collection of socio-demographic and clinical data are also described elsewhere [19]. All the enrolled children had a capillary or venous blood sample to measure CRP and hemoglobin instantly. Plasma specimens at baseline were obtained in 430 of the patients, but there was too little plasma from three of the children to analyze cytokines. End-study blood samples were taken in 350 children and obtained after 14 days in 182 children, after 45 days in 83 children, and after 90 days in 85 children (Supplementary Materials Figure S1). These time points were selected based on other predefined outcomes of the study [22]. The specimens were transported on dry ice and kept frozen at −70 °C until analysis. Plasma zinc concentration was determined by inductively coupled plasma mass spectrometry (PlasmaQuad 3; VG Elemental, Winsford, Cheshire, UK). Hemoglobin was measured by Hemocue (Hemocue, Vedbæk, Danmark). Cytokine analyses in plasma were performed at Broegelmanns Research Laboratory, University of Bergen, Norway using magnetic bead-based multiplex immunoassay for Luminex 100 system (Luminex Corp. Austin, TX, USA) using the StarSTation v3 (Applied Cytometry Systems, Dinnington, UK) software. The kit used was the premade “Bio-Plex Pro™ Human Cytokine Standard 27-Plex, Group I-kit” from BioRad (BioRad Laboratories, Inc. Life Science research, Hercules, CA, USA). Data from the reactions were acquired using the Luminex reader, while a digital processor managed the data output and the Luminex software returned data as median fluorescence intensity (MFI) and concentration (pg/ml). The following cytokines were included in the kit: Interleukin (IL)-1β, IL-1ra, IL-2, IL-4, IL-5, IL-6, IL-7, IL-8, IL-9, IL-10, IL-12 (p70), IL-13, IL-15, IL-17A, basic fibroblast growth factor (basic-FGF), eotaxin, granulocyte colony-stimulating factor (G-CSF), granulocyte-macrophage colony-stimulating factor (GM-CSF), Interferon-gamma (IFN-γ), Interferon-inducing protein-10 (IP-10), MCP-1, macrophage inflammatory protein 1-alpha (MIP-1α), macrophage inflammatory protein 1-beta (MIP-1β), regulated on activation, normal T-cell expressed and secreted (RANTES), tumor necrosis factor-alpha (TNF-α), platelet-derived growth factor subunit B (PDGF-BB), and vascular endothelial growth factor (VEGF). The serum concentration of the analytes was determined using known standards included in the kits, and the calculation of 5-parametric standard curves (StarSTation v.3, Applied Cytometry, Dinnington, UK) [23]. Vitamin D in plasma was analyzed at the Laboratory of Clinical Chemistry, Innlandet Hospital Trust, Lillehammer, Norway using a Vitamin D total kit from Roche on Cobas. This is a protein-binding method binding both 25(OH)D_2_ and 25(OH)D_3_.

### 2.3. Statistics

For description of demographic and clinical characteristics, inflammatory markers and vitamin D status, mean ± SD, median (IQR), or proportions (%) were estimated separately for children with non-severe and severe pneumonia. Spearman’s correlation was used to estimate correlations between inflammatory markers and 25(OH)D concentrations. Non-parametric testing was chosen because of the distribution of the inflammatory markers. A two-sample *t*-test with equal variances was used for estimating differences in 25(OH)D concentrations between non-severe and severe pneumonia groups at baseline and after recovery. We also used generalized additive models (GAM) in the statistical software R version 3.16 (R Foundation for Statistical Computing, Vienna, Austria) to explore adjusted nonlinear associations between age or breastfeeding frequency and 25(OH)D concentration [24].

We identified predictors of 25(OH)D concentration in multiple linear regression models using a step-wise manual selection process [25]. The following candidate variables were included in this process: age of the child (at the time of blood sampling), gender, *z*-scores weight for length as continuous variables, clinical severity based on non-severe/severe groups, CRP, breastfeeding frequency, mother’s and father’s literacy and occupation, ownership of agricultural land, number of children, adults, and rooms in the household, indoor tobacco smoke, time until recovery, treatment failure, zinc or placebo-treatment, solar radiation, and inflammatory markers. Ownership of land may be a proxy of socioeconomic status, but was also chosen as a candidate variable because of the possible impact of agricultural work on sun exposure. We used two different approaches to predict 25(OH)D concentrations, (1) using only baseline data, i.e., when the children were acutely ill with ALRI; and (2) using data from both the baseline and the second sample i.e., two samples from each child when he or she was ill and 14, 45, or 90 days later. In the latter approach, we could compare the concentrations at baseline and recovery in the same children, taking age into account. In these models, we adjusted the CI and *p* values for lack of independence between the two different measurements for each child. The values of the 27 different cytokines/chemokines and growth factors were log-transformed when included in the linear models.

To estimate the influence of sunlight on 25(OH)D levels, we used monthly global solar radiation as given in the article by Adhikari et al. from the Institute of Engineering, Tribhuvan University, Kathmandu, Nepal. This article provides estimated data for global solar radiation in MJ/m^2^/day for the Kathmandu area between 2007 and 2012, using models based on the available parameters sunshine duration, maximum and minimum temperature, relative humidity, rainfall, and geographical location [26]. The half-life of 25(OH)D of approximately three weeks will, however, cause a lag for the effect on vitamin D_3_ synthesis, so in order to express the association between global solar radiation and 25(OH)D we used the mean of the last three months’ global solar radiation (Supplementary Materials Figure S2). A *p* value of 0.05 was considered significant in the final model. Recovery samples were divided into second sample times of 14, 45, and 90 days.

## 3. Results

### 3.1. Child Characteristics

The demographic, clinical characteristics, inflammatory markers, and vitamin D status of the children are described in Table 1.

### 3.2. Concentrations and Correlations of 25(OH)D and Inflammatory Markers

Of the 412 children with reliable 25(OH)D concentrations, 85.2% were vitamin D sufficient (25(OH)D ≥ 50 nmol/L), 10.4% were insufficient (30–49 nmol/L), and 4.4% were deficient (<30 nmol/L) (Table 1). The concentrations of the inflammatory markers IL-1β, IL-ra, IL-6, IL-8, IL-10, G-CSF, IP-10, and VEGF were higher at baseline than at recovery (Supplementary Materials Figure S3). Plasma concentrations of 25(OH)D were slightly higher at baseline compared to after recovery (14, 45, and 90 days after the acute episode), with mean concentrations of 84.4 and 80.6 nmol/L, respectively (Table 2).

There were also weak positive correlations between vitamin D concentration and IL-6, IL-15, eotaxin, and GM-CSF in the baseline samples and weak positive correlations for CRP, IL-4, IL-6, IL-9, IL-15, eotaxin, G-CSF, GM-CSF, and IP-10 in the recovery samples (Table 3).

**Figure 1:**
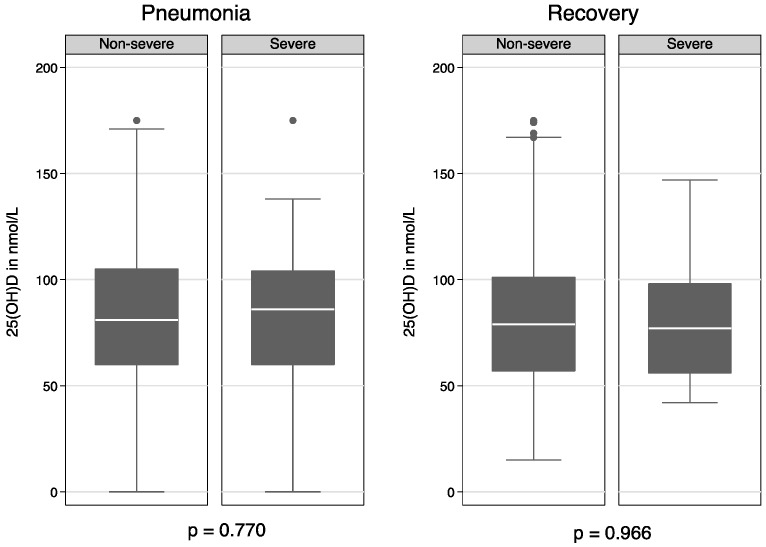
25(OH)D concentration for non-severe and severe pneumonia at enrollment and after recovery. Differences in means are estimated by two-sample *t*-test with equal variances.

The 25(OH)D concentration was similar in the non-severe and severe groups both at baseline (83.4 ± 34.7 vs. 85.5 ± 32.1) and at recovery (80.6 ± 35.4 vs. 80.7 ± 27.0), respectively (Figure 1).

### 3.3. Predictors of 25(OH)D Concentration

**Figure 2:**
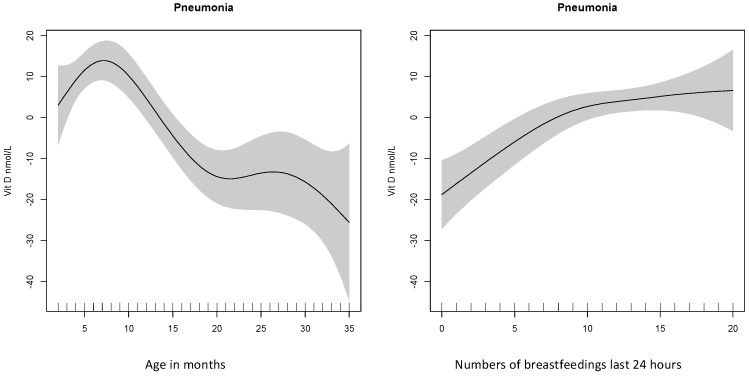
Vitamin D concentration according to age and breastfeeding frequency. Vitamin D concentration in nmol/L according to age and breastfeeding frequency by baseline; vitamin D concentration centered around its mean. The graphs showed a similar picture for recovery samples.

The variables age, breastfeeding frequency, and ownership of land were positively associated with 25(OH)D concentration at baseline and explained 26% of its variability (Table 4 and Figure 2).

Age was the only variable associated with 25(OH)D concentration in the recovery samples when analyzing these data separately (Supplementary Materials Table S1). Relevant variables that were not associated in the multiple regression models were gender, weight for length *z*-score, solar radiation, severity of pneumonia, treatment with zinc or placebo, socioeconomic variables like number of children/adults/rooms in the household, and indoor tobacco smoking. None of the inflammatory markers were associated with 25(OH)D concentration in the multiple regression models. Age and ownership of agricultural land could explain 24% of the variability in the model including both 1st and 2nd sample (Table 5).

The 25(OH)D concentration was significantly different from the baseline 14 and 90 days after the pneumonia episode, but these differences were substantially attenuated and became insignificant when adjusting for age.

## 4. Discussion

To elucidate if plasma-25(OH)D changes during infection or by inflammation, we measured 25(OH)D concentration and inflammatory markers during acute illness and after recovery in children with non-severe and severe pneumonia. We also explored the associations between clinical and inflammatory markers and plasma 25(OH)D concentration. There were no differences in plasma-25(OH)D between the groups of non-severe and severe pneumonia either during or after infection, and there were no differences between baseline and recovery 25(OH)D when adjusting for age. Age of the child, ownership of agricultural land in the family, and breastfeeding frequency were significant predictors of 25(OH)D concentration, while the severity of infection and inflammatory markers were not.

In our study we observed a slightly increased 25(OH)D concentration during the acute phase compared to after recovery in the crude analysis. As expected, the acute phase was characterized by a significant increase in the concentration of several inflammatory markers. However, there were positive correlations between the inflammatory markers and 25(OH)D concentration both at baseline and after recovery, and there was no difference in the 25(OH)D concentration between non-severe and severe pneumonia cases, which is contrary to the hypothesis of 25(OH)D as a negative acute phase reactant. Vitamin D/vitamin D receptor (VDR) signaling is involved in several immune modulating actions of adaptive and innate immune cells [6,7,8,9]. The weak positive correlations between vitamin D concentration and some of the inflammatory markers were not significant in the multiple models, however, and are therefore difficult to interpret. When adjusting for age there also was no significant difference between plasma-25(OH)D measured during pneumonia and after recovery. The finding of similar 25(OH)D concentrations in baseline and recovery samples is consistent with the study of 30 children with bacterial infections [18], where no differences was found during acute illness compared to four weeks after the infection.

Age was the main predictor for vitamin D status and was negatively associated with the 25(OH)D concentration, both for the baseline and recovery samples, and could explain the slight decrease in 25(OH)D concentration from enrollment to recovery since all the children were up to three months older when the second sample was taken. There could be several explanations for this age effect. There is no current recommendation of vitamin D supplementation for infants in Nepal and the use of supplements and infant formulas is uncommon [27]. However, data on the use of infant formulas and other supplements are lacking, and infant formula as a possible contributor to the higher levels in the youngest children cannot be ruled out. In Bhaktapur there is also a local tradition of giving oil massages to the naked infant while sunbathing, and outdoor breastfeeding habits have previously been discussed as a possible reason for replete vitamin D status in young Nepalese children [28]. An interesting finding in our study was that breastfeeding frequency at baseline was positively associated with plasma-25(OH)D, which could, in part, reflect these sunbathing habits. Recent studies also suggest that breast milk is adequate in vitamin D when the mother has sufficient circulating concentrations of vitamin D_2_ and/or vitamin D_3_ [29,30,31], and the vitamin D_3_ content of the breast milk is also reported to increase many times compared to 25(OH)D_3_ in the mother’s serum after sun exposure [32]. The positive association with breastfeeding frequency could therefore be due to direct production in the child’s skin during outdoor breastfeeding and oil massage and/or maternal transfer of vitamin D metabolites through the breast milk. The positive association with ownership of agricultural land could also fit into this picture, with a possibility of more sun exposure for both mother and child in agricultural families.

There are some limitations to the study. Pre-infection vitamin D status would have been better than post-infection samples, but would have required another study design and a larger number of participants. Inclusion of children with more severe disease, measuring other inflammatory markers, would maybe have yielded other results. With only 43 patients admitted to hospital for intravenous antibiotic treatment and other supportive therapy, significant hemodilution seems unlikely at baseline, but significant dehydration could also mask a lower plasma-25(OH)D by baseline. We would need more complete data on hydration status to further elucidate its possible role. Finally, similar to most commercial methods for measuring 25(OH)D concentration, including commercial HPLC-MS/MS, the kit used in this study did not separate the C3-epimeric forms of 25(OH)D [33]. An overestimation of 25(OH)D, especially in infants, can therefore not be excluded [34]. Although the importance of C3 epimers is still not fully elucidated, the presence in infants has been reported to amount for 6%–60% of the vitamin D status [35], and should be acknowledged in further studies. The strengths of the study are the large population size and the well-characterized illness, both clinically and biochemically.

## 5. Conclusions

In conclusion, our findings indicate that we do not need to take inflammation or infection into account when interpreting the concentration of plasma-25(OH)D. Age was the strongest predictor of vitamin D status, and differences in age explained the slight difference in 25(OH)D concentration between baseline and recovery.

## Figures and Tables

**Table 1:** Baseline characteristics of Nepalese children with community-acquired pneumonia, by severity.

Child Characteristics		Non-Severe		Severe	
		*n*	Value	*n*	Value
**Demographic data**					
Age of child (months)	mean ± SD	387	14.1 ± 8.5	43	7.7 ± 7.4
Boys	*n* (%)	387	205 (53.0)	43	26 (60.5)
Breastfed—yes	*n* (%)	387	367 (94.8)	43	41 (95.3)
Number of breast feedings past 24 h	mean ± SD	387	10.6 ± 5.6		11.4 ± 6.0
Family ownership of land	*n* (%)	387	185 (47.8)	43	20 (46.5)
Living in nuclear family (*)	*n* (%)	387	188 (48.6)	43	24 (55.8)
Number of family members	mean ± SD	387	6.2 ± 3.3	43	5.5 ± 3.0
Indoor smoking	*n* (%)	386	248 (64.2)	42	22 (52.4)
*Z*-score weights for age	mean ± SD	387	−0.8 ± 1.1	43	−0.8 ± 1.3
*Z*-score length/height for age	mean ± SD	387	−1.1 ± 1.2	43	−0.7 ± 1.5
*Z*-score weight for length/height	mean ± SD	386	−0.3 ± 1.0	43	−0.5 ± 1.1
**Clinical characteristics**					
Axillary temperature (°C)	mean ± SD	387	37.3 ± 0.9	43	37.3 ± 0.7
Duration of cough (days)	mean ± SD	387	3.2 ± 2.0	43	3.1 ± 1.8
Duration of difficulty breathing (days)	mean ± SD	387	1.7 ± 1.9	43	1.8 ± 1.5
Duration of fever (days)	median (IQR)	387	2 (1–3)	43	2 (1–3)
Respiratory rate/min	mean ± SD	387	53 ± 6.7	43	66.0 ± 9.2
Presence of lower chest indrawing (LCI)	*n* (%)	387	0 (0.0)	43	42 (97.7)
SpO_2_ (%)	median (IQR)	387	93.5 (91.5–96.5)	43	91.5 (90.5–95.0)
Hypoxia (SpO_2_ < 90%)	*n* (%)	387	3 (0.8)	43	9 (20.9)
Time until recovery (days) (**)	median (IQR)	387	3 (2–5)	43	6 (4–8)
CRP (mg/L)	median (IQR)	387	12 (0–26)	43	25 (0–50)
CRP > 40 (mg/L)	*n* (%)	387	48 (12.4)	43	14 (32.6)
Hemoglobin (g/dL)	mean ± SD	387	11.2 ± 1.3	43	10.7 ± 1.5
**Vitamin D status**					
Plasma-25(OH)D (nmol/L)	mean ± SD	373	83.4 ± 34.7	40	85.6 ± 32.1
Plasma-25(OH)D ˂ 30 (nmol/L)	*n* (%)	373	20 (5.4)	40	1 (2.5)
Plasma-25(OH)D 30–49 (nmol/L)	*n* (%)	373	42 (11.3)	40	1 (2.5)
Plasma-25(OH)D ≥ 50 (nmol/L)	*n* (%)	373	312 (83.6)	40	39 (97.5)

* Nuclear family = children living together with their parents. ** Time until recovery from non-severe pneumonia. Demographic and clinical data previously published in: doi:10.1371/journal.pone.0138978.

**Table 2:** Mean 25(OH)D concentration during pneumonia and after recovery in Nepalese children.

	During Pneumonia			After Recovery		
	*n*	Mean (*)	SD	*n*	Mean (*)	SD
All children regardless of time for 2nd sample	412	84.4	33.6	343	80.6	35.4
Children with 2nd sample after 14 days	196	82.9	32.9	180	80.0	32.5
Children with 2nd sample after 45 days	102	96.7	35.8	82	90.8	33.3
Children with 2nd sample after 90 days	114	76.1	29.7	81	71.6	38.4

* 25(OH)D in nmol/L.

**Table 3:** Spearman’s correlations between vitamin D concentration and demographic data, clinical characteristics, and inflammatory markers by baseline and after recovery.

Inflammatory Markers	Baseline (VITD1) (*n =* 407 or 408)		After Recovery (*) (VITD2) (*n =* 338 or 339)	
	*r_s_*	*p*	*r_s_*	*p*
CRP	0.035	0.484	0.202	**<0.001**
IL-1β	0.095	0.056	0.017	0.753
IL-1ra	0.011	0.824	0.061	0.265
IL-4	0.062	0.213	0.111	**0.041**
IL-6	0.133	**0.007**	0.160	**0.003**
IL-8	−0.040	0.424	0.006	0.912
IL-9	0.085	0.087	0.213	**<0.001**
IL-10	−0.043	0.382	0.027	0.622
IL-15	0.209	**<0.001**	0.222	**<0.001**
eotaxin	0.124	**0.013**	0.121	**0.027**
Basic-FGF	0.020	0.690	0.080	0.141
G-CSF	0.070	0.156	0.123	**0.024**
GM-CSF	0.161	**0.001**	0.205	**<0.001**
TNF-α	−0.033	0.504	0.058	0.284
IP-10	−0.046	0.357	0.122	**0.025**
VEGF	0.018	0.715	0.035	0.523

Continuous demographic and lab data and inflammatory markers showing significant elevation in baseline samples compared to recovery sample and/or significant differences between groups of clinical severity at baseline were run in a Spearman’s rank correlation. (*r_s_* 0.1–0.29 = weak correlation, *r_s_* 030–0.50 = moderate correlation, *r_s_* > 0.50 = strong correlation); * Samples taken after 14, 45, or 90 days.

**Table 4:** Multiple regression model with predictors for plasma 25(OH)D concentrations in Nepalese children with community-acquired pneumonia.

25(OH)D during Pneumonia	Crude (*n =* 412)			Adjusted (*n =* 411)			
	Coeff	95% CI	*p*	Coeff	95% CI	*p*	Beta
Age in months	−1.9	−2.2, −1.6	**<0.001**	−1.5	−1.9, −1.1	**<0.001**	−0.4
No. of breast feedings last 24 h	2.3	1.8, 2.8	**<0.001**	1.1	0.5, 1.7	**<0.001**	0.2
Ownership of agricultural land (*)	−7.4	−13.9, −0.9	**0.025**	−7.6	−12.5−1.3	**0.016**	−0.1

Sex, *z*-scores weight for length, clinical severity based on non-severe/severe groups, CRP, mother’s and father’s literacy and occupation, number of children/adults/rooms in the household, indoor tobacco smoke, time until recovery, treatment failure, zinc or placebo treatment, solar radiation, and inflammatory markers were not significantly associated with 25(OH)D concentration. Adjusted R-squared = 0.26. Beta: standardized regression coefficient. * Coded yes = 1, no = 2.

**Table 5:** Multiple regression model of predictors for plasma 25(OH)D concentrations in Nepalese children during and after recovery from community-acquired pneumonia.

25(OH)D	Crude			Adjusted			
	Coeff	95% CI	*p*	Coeff	95% CI	*p*	Beta
Baseline plasma sample (ref)							
14 days plasma sample	−4.7	−8.8, −0.6	**0.026**	−3.0	−6.7, 0.7	0.117	−0.0
45 days plasma sample	6.1	−0.7, 12.8	0.077	2.3	−3.9, 8.5	0.469	0.0
90 days plasma sample	−12.6	−21.7, −3.5	**0.007**	−2.1	−10.4, 6.1	0.615	−0.0
Age in months	−1.9	−2.2, −1.6	**<0.001**	−1.9	−2.2, −1.6	**<0.001**	−0.5
Ownership of agricultural land	−6.0	−12.0, 0.6	0.076	−5.8	−11.6, −0.0	**0.048**	−0.1

25(OH)D in first and second samples: Number of obs = 751 Adjusted R-squared = 0.24 Adjusted for repeated measurements in each child. Beta: standardized regression coefficient. Sex, *z*-scores weight for length, clinical severity based on non-severe/severe groups, CRP, mother’s and father’s literacy and occupation, number of children/adults/rooms in the household, indoor tobacco smoke, time until recovery, treatment failure, zinc or placebo treatment, solar radiation, and inflammatory markers were not significantly associated with 25(OH)D concentration.

## Supplementary Materials

The following are available online at http://www.mdpi.com/2072-6643/9/1/52/s1, Table S1: Multiple regression model with predictors for plasma 25(OH)D concentrations in Nepalese children after recovery from community-acquired pneumonia, Figure S1:The patient flow in the study. Out of the 2628 patients, 430 were randomly selected to measure plasma cytokines and 25(OH)D, Figure S2: Estimated average global solar radiation for current and previous two–four months. Dotted line: Global solar radiation for the last month. Dashed line: Mean global solar radiation for the two last months. Black solid line: Mean global solar radiation for the three last months. Grey solid line: Mean global solar radiation for the four last months, Figure S3: Differences in concentrations of inflammatory markers during pneumonia (1) and after recover (2). 25(OH)D-concentration by baseline (1) and after recovery (2). Differences in means are calculated by using rank sum.
