# Supplementary material for: 25-Hydroxy-Vitamin D Concentration Is Not Affected by Severe or Non-Severe Pneumonia, or Inflammation, in Young Children
Source: Nutrients. 2017 Jan 17;9(1):52. doi: 10.3390/nu9010052 (PMC5295096; doi:10.3390/nu9010052)
Supplement: Supplementary file 1 [file nutrients-09-00052-s001.docx]

**Supplementary Materials: 25-Hydroxy-Vitamin D Concentration Is Not Affected by Severe or Non-Severe Pneumonia, or Inflammation in Young Nepalese Children**

Johanne Haugen, Ram K. Chandyo, Manjeswori Ulak, Maria Mathisen, Sudha Basnet,
Karl A. Brokstad, Palle Valentiner-Branth, Prakash S. Shrestha and Tor A. Strand

**Table S1.** Multiple regression model with predictors for plasma 25(OH)D concentrations in Nepalese children after recovery from community-acquired pneumonia.

| **25(OH)D in Second Sample** | **Crude** | | | **Adjusted** | | | |
| --- | --- | --- | --- | --- | --- | --- | --- |
|  | **Coeff** | **95% CI** | ***p*** | **Coeff** | **95% CI** | ***p*** | **Beta** |
| 14 days ref |  |  |  |  |  |  |  |
| 45 days | 10.8 | 1.9, 19.8 | **0.018** | 5.3 | −2.6, 13.3 | 0.185 | 0.1 |
| 90 days | −8.4 | −17.4, 0.6 | 0.068 | 0.15 | −7.9, 8.2 | 0.971 | 0.0 |
| Age in months by second sample | −2.0 | −2.4, −1.7 | **<0.001** | −2.0 | −2.4, −1.6 | **<0.001** | −0.5 |

Sex, *z*-scores weight for length, clinical severity based on non-severe/severe groups, CRP, mother´s and father´s literacy and occupation, number of children/adults/rooms in the household, indoor tobacco smoking, time until recovery, treatment failure, zinc or placebo treatment, solar radiation, ownership of agricultural land, and inflammatory markers were not significantly associated with 25(OH)D concentration. Adjusted R-squared = 0.25. Beta: standardized regression coefficient.

**Figure S1**. The patient flow in the study. Out of the 2628 patients, 430 were randomly selected to measure plasma cytokines and 25(OH)D.

**Figure S2.** Estimated average global solar radiation for current and previous two–four months. Dotted line: Global solar radiation for the last month. Dashed line: Mean global solar radiation for the two last months. Black solid line: Mean global solar radiation for the three last months. Grey solid line: Mean global solar radiation for the four last months.

**Figure S3.** Differences in concentrations of inflammatory markers during pneumonia (1) and after recover (2). 25(OH)D-concentration by baseline (1) and after recovery (2). Differences in means are calculated by using rank sum.
